# Pseudotumoral tracheobronchial amyloidosis mimicking asthma: a case report

**DOI:** 10.1186/1752-1947-6-40

**Published:** 2012-01-30

**Authors:** Mounia Serraj, Imane Kamaoui, Kawtar Znati, Salma Kouara, Ferdaous Sahnoune, Bouchra Amara, Mohammed El Biaze, Siham Tizniti, Afaf Amarti, Mohammed Chakib Benjelloun

**Affiliations:** 1 Department of Lung Disease, Hassan II University Hospital of Fez, Fez, Morocco; 2 Department of Radiology, Hassan II University Hospital of Fez, Fez, Morocco; 3 Laboratory of Pathology, Hassan II University Hospital of Fez, Fez, Morocco

## Abstract

**Introduction:**

Tracheobronchial amyloidosis is an uncommon localized form of amyloidosis that can simulate a tracheal tumor. Clinical signs are not specific and the diagnosis is rarely given before performing a bronchoscopy with multiples biopsies.

**Case presentation:**

We report the case of a 60-year-old Moroccan woman, complaining of dyspnea and wheezing for three years, who was treated at our institution for management of severe asthma. A bronchoscopy revealed a tumor formation of her trachea; multiples biopsies were performed and a diagnosis made of amyloid light-chain amyloidosis. She successfully received an endoscopic resection.

**Conclusion:**

This case highlights the importance of routinely carrying out an endoscopy in any patient complaining of atypical bronchial symptoms or with uncontrolled asthma. Tracheal amyloidosis is a rare disease, confirmed by histological examination of bronchial biopsies, and the treatment of choice is based on the bronchoscopic resection.

## Introduction

Amyloidosis refers to the process of abnormal deposition of protein fibrils in extracellular tissue. Tracheobronchial amyloidosis is one of the localized variants of amyloidosis. With only around one hundred cases reported in the literature, it can be qualified as a rare disease. In this case report, we study its clinical presentation, histological features and the therapeutic options.

## Case presentation

A 60-year-old Moroccan woman, treated for hypertension, complained of persistent cough, chest tightness and wheezing. Three years previously she was diagnosed with bronchial asthma by her family physician. This diagnosis of asthma was retained for episodes of shortness of breath with recurrent respiratory wheeze. A chest radiograph appeared normal. Spirometry was performed and showed an obstructive syndrome, but its interpretation was difficult due to the poor cooperation of our patient. The flow volume curve was considered invalid. She was treated for probable asthma with inhaled corticosteroids. Despite her adherence to the treatment, her symptoms worsened with a progressive decrease in exercise tolerance, increased wheezing episodes and shortness of breath. She was then referred to our hospital with uncontrolled asthma.

A physical examination revealed a woman in good condition, a respiratory rate of 24 cycles per minute and no rales. The rest of the examination was normal. Her laboratory tests were unremarkable.

A chest X-ray showed a right laterotracheal opacity at the cervicothoracic junction. There was also a reduction of tracheal clarity, consistent in several chest radiographs.

**Figure 1:**
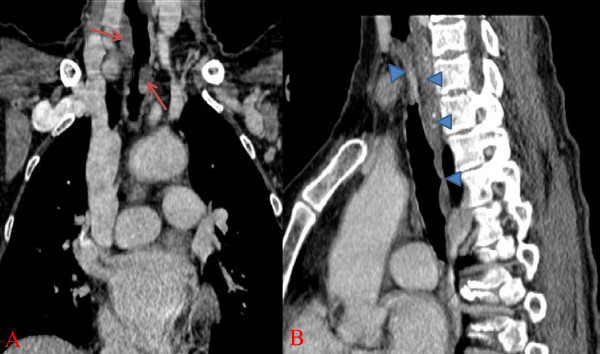
**Tracheal computed tomography**. A circumferential tracheal tumor caused a reduction of her caliber.

Computed tomography (CT) of her chest with a three-dimensional reconstruction confirmed the presence of thickening of her tracheal wall tissue; it was circumferential and irregular, causing a significant reduction in her tracheal caliber (Figure 1a, b). The lesion had intimate contact with her esophagus without evidence of invasion.

**Figure 2:**
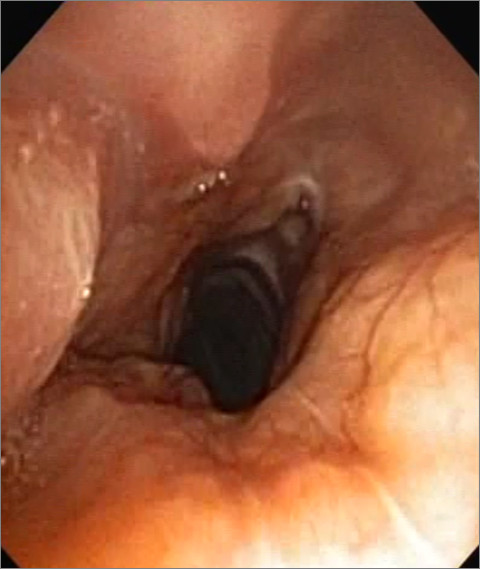
**Bronchoscopy findings**. Circumferential and irregular tracheal tumor with a significant reduction of her tracheal caliber.

Bronchoscopy was performed and showed a yellowish tumor formation just below her vocal cords, extending along the entire height of her trachea and reducing its diameter by more than 50%. This lesion was larger in the posterior wall and covered the side walls of her trachea. The carina was normal (Figure 2).

**Figure 3:**
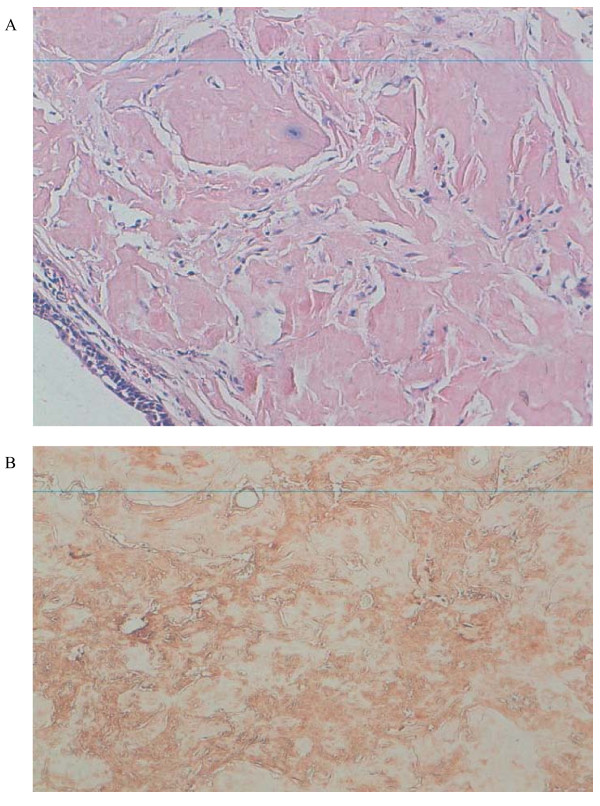
**Tracheal biopsy and histology findings**. **(a) **A tracheal biopsy revealed a chorion occupied by massive amyloid deposits. (b) Intense staining by anti-lambda light chain antibodies confirming the diagnosis of an amyloid light-chain amyloid tumor.

The tracheal biopsies revealed a chorion occupied by massive amyloid deposits (Figure 3a). These deposits showed a green birefringence under crossed polarized light after Congo red staining. An immunohistochemical analysis showed intense staining by anti-lambda light chain antibodies confirming the diagnosis of an amyloid light-chain (AL) amyloid tumor (Figure 3b).

A search for systemic disease or monoclonal gammopathy remained negative. This review included immunoelectrophoretic analysis of her plasma and urine, salivary gland and rectal biopsies and X-rays of flat bones to look for myeloma.

In conclusion, our patient had a localized amyloidosis in her respiratory tract, revealed by a false asthma. A rigid endoscopy was performed to permeabilize her trachea. Three sessions were required to obtain a satisfactory tracheal size and resolution of symptoms. The last endoscopy was complicated by a wound in the mucosa with iatrogenic pneumomediastinum. A CT scan with ingestion of hydrosoluble product showed no esophageal injury. Our patient had no symptoms after this incident.

After 12 months, our patients respiratory symptoms have disappeared and endoscopic lesions are stable.

## Discussion

Amyloidosis is a generic term for heterogeneous disorders associated with the deposition of proteins in abnormal fibrillar form [1,2]. Amyloidosis can be hereditary or acquired, localized or systemic and is potentially lethal. The deposits accumulate in the extracellular space, progressively disrupting tissue architecture, and can impair organ function. Solitary amyloid deposits in the tracheobronchial tree or pulmonary parenchyma are unusual manifestations of primary amyloidosis [3,4].

Amyloid deposition may occur in association with inflammatory or neoplastic conditions or develop as part of an immunoglobulin (Ig) disorder.

Virchow first described amyloidosis involving the lungs in 1857 [3]. The first case of amyloidosis confined to the lower respiratory tract was described by Lesser in 1857 based on an autopsy study [5]. Amyloidosis may involve the lungs as part of a systemic process or may be confined solely to the lungs. Localized pulmonary amyloidosis may involve either lung parenchyma or the airways.

The most frequent types of protein amyloidosis are AL (primary) and AA (secondary) types. AL amyloidosis results from plasma cell dyscrasia that produces monoclonal light-chain Igs, whose fragments deposit as this protein. AA amyloidosis occurs in chronic diseases (for example, rheumatoid arthritis and chronic infection).

Localized AL amyloidosis is most often identified in the upper respiratory, urogenital and gastrointestinal tracts, the skin and the orbit [1].

Tracheobronchial amyloidosis is among the localized variants of amyloidosis. Up until 2004, fewer than 150 cases were reported; about 86 cases were published in the literature as of 2009 in China. Most of the reports involved were a single case or small case series. Only a few were randomized control clinical studies. Tracheobronchial amyloidosis is characterized by the deposition of amyloid material as submucosal plaques and/or polypoid tumors in the airways [6-8], which may be localized, diffuse or multifocal. The endobronchial form is not associated with systemic amyloidosis [7-9].

The morbidity and mortality of tracheobronchial amyloidosis directly correspond to the quantity of amyloid infiltrating the airways. Death is a result of the progressive bronchial obstruction and respiratory failure. This condition can be completely asymptomatic or be revealed by dyspnea, wheezing, hemoptysis, recurrent pneumonia, cough and atelectasis [7-11]. Proximal or upper tracheal disease presents with various degrees of obstruction; mid- or distal tracheal, main bronchial disease involves lobar collapse or recurrent parenchymal infections, severe obstruction of the main bronchi and respiratory failure. Distal airway disease presents with recurrent pneumonia, cough and bronchiectasis. However, the symptoms are usually nonspecific, mimicking common respiratory conditions such as bronchial asthma (Table 1; [12-15]).

**Table 1:** Symptoms of tracheobronchial amyloidosis in the literature.

Reference	Number of patients	Mean age	Common symptoms			
			
			Wheezing, asthma	Pneumonia	Hemoptysis	Cough, sputum
[12] 1970 to 2009	64	49.0	5	3	0	56

[13] 1973 to 1999	17	56.0	2	0	4	16

[14] 1983 to 2002	32 with 28 with tuberculosis	52.0	0	6	7	20

[15] 1970 to 1985	21 with 5 with tuberculosis	59.8	0	3	2	5

The diagnosis of amyloidosis usually requires histological confirmation. Congo red staining that produces green birefringence under crossed light remains the gold standard [16]. Positive histology results for amyloid must be followed-up by immunohistochemical analysis to determine the fibril type.

In amyloidosis of the respiratory tract, a complete evaluation must be done. It should include radiography, CT scanning, endoscopy and respiratory function tests. Evidence of systemic disease should be sought clinically, including examination and immunofixation of serum and urine for a monoclonal protein.

Treatment strategies are variable depending on the degree of obstruction (Table 2). No treatment with simple monitoring is proposed in asymptomatic patients, whereas more aggressive local and/or systemic therapy is undertaken when the obstruction is significant and the patient symptomatic [11,17,18]. Bronchoscopic debridement with forceps debulking remains the standard therapeutic approach to upper and mid-airway disease. Repeated debulking is commonly required [11,19,20]. A silicone stent with local resection using a carbon dioxide laser or yttrium aluminum garnet laser have also been reported [21]. Repeated bronchoscopic intervention is thought to be preferable and safer than open surgery [10]. Isolated cases report the use of radiotherapy in the treatment of localized tracheobronchial amyloidosis [22,23].

**Table 2:** Endoscopic features and treatment.

Reference	Endoscopic appearances			Endoscopic treatment			Other treatment		
	
	Submucosal plaques	Pseudotumor appearance	Wall thickening	Laser resection	Mechanical resection (debulking)	Stent	Supervision	Pharmacological	External radiation therapy
[12] 1970 to 2009 n = 64	-	22	17	17	-	0	15	19	4

[13] 1973 to 1999 n = 17	10	0	7	3	4	0	10	2	1

[14] 1983 to 2002 n = 28	14	9	9	27	-	3	-	-	1

[15] 1970 to 1985 n = 5	3	2	0	-	-	-	4	1	-

## Conclusion

Patients with tracheobronchial amyloidosis have symptoms similar to those caused by various airway disorders. Early recognition and prompt intervention are crucial to avert complications, especially respiratory failure. However, new approaches to treatment are required and agents that could stabilize amyloid precursor proteins in their normal conformation, inhibit fibril formation and/or enhance fibril degradation are currently being sought.

## Consent

Written informed consent was obtained from the patient for publication of this case report and any accompanying images. A copy of the written consent is available for review by the Editor-in-Chief of this journal.

## Competing interests

The authors declare that they have no competing interests.

## Authors' contributions

MS conceptualized the case study, gathered the data and wrote the manuscript. IK interpreted the data, performed the radiology and revised the manuscript. KZ and AA performed the histological evaluation and interpretation of the data. SK and FS acquired the data. BA, MB, ST and MCB gave final approval for publication. All authors read and approved the final manuscript.
